# The Polymorphism in the Promoter of HSP70 Gene Is Associated with Heat Tolerance of Two Congener Endemic Bay Scallops (*Argopecten irradians irradians* and *A. i. concentricus*)

**DOI:** 10.1371/journal.pone.0102332

**Published:** 2014-07-16

**Authors:** Chuanyan Yang, Lingling Wang, Jingjing Wang, Qiufen Jiang, Limei Qiu, Huan Zhang, Linsheng Song

**Affiliations:** 1 Key Laboratory of Experimental Marine Biology, Institute of Oceanology, Chinese Academy of Sciences, Qingdao, China; 2 University of Chinese Academy of Sciences, Beijing, China; 3 Qingdao Agricultural University, Qingdao, China; Uppsala University, Sweden

## Abstract

**Background:**

The heat shock protein 70 (HSP70) is one kind of molecular chaperones, which plays a key role in protein metabolism under normal and stress conditions.

**Methodology:**

In the present study, the mRNA expressions of HSP70 under normal physiological condition and after acute heat stress were investigated in gills of two bay scallop populations (*Argopecten irradians irradians* and *A. i. concentricus*). The heat resistant scallops *A. i. concentricus* showed significantly lower basal level and higher induction of HSP70 compared with that of the heat sensitive scallops *A. i. irradians*. The promoter sequence of HSP70 gene from bay scallop (AiHSP70) was cloned and the polymorphisms within this region were investigated to analyze their association with heat tolerance. Totally 11 single nucleotide polymorphisms (SNPs) were identified, and four of them (−967, −480, −408 and −83) were associated with heat tolerance after HWE analysis and association analysis. Based on the result of linkage disequilibrium analysis, the *in vitro* transcriptional activities of AiHSP70 promoters with different genotype were further determined, and the results showed that promoter from *A. i. concentricus* exhibited higher transcriptional activity than that from *A. i. irradians* (*P*<0.05).

**Conclusions:**

The results provided insights into the molecular mechanisms underlying the thermal adaptation of different congener endemic bay scallops, which suggested that the increased heat tolerance of *A. i. concentricus* (compared with *A. i. irradians*) was associated with the higher expression of AiHSP70. Meanwhile, the −967 GG, −480 AA, −408 TT and −83 AG genotypes could be potential markers for scallop selection breeding with higher heat tolerance.

## Introduction

Heat shock proteins (HSPs) are a suite of highly conserved proteins with varying molecular weight and play important roles in protecting organisms against various stressors. Among them, HSP70s is a large HSP family with molecular weight of 70 kDa. They can not only act as molecular chaperones, functioning on modulating stress response and decreasing cellular damage induced by environment [1] but also play a role in immune responses and cancer immunity [2]–[4]. In the past few decades, the relationship between the expression level of HSP70 and heat tolerance has been well studied. For example, the heat tolerance of *Arabidopsis* reduced in HSP70-knockout plants [5] and enhanced in HSC70-1 -overexpressed plants [6], and the heat tolerance of *Drosophila* was positively associated with the expression of HSP70 [7]. Moreover, many studies have demonstrated that the heat tolerance of a species or genus could be enhanced by increasing the copy number of the HSP70 genes [8]–[10]. For example, the high heat tolerance of *Crassostrea gigas* was believed to associate with the remarkably genomic expansion and massive up-regulation of HSP70 genes [10]. However, there are so many species with different heat tolerance preserving the same number of HSP70 copies and exhibiting significantly different expression patterns of HSP70 after heat stress [11]–[14], indicating the efficiency variance and sequence polymorphisms in the promoter of HSP70 genes.

During the last decade, a certain number of polymorphisms in HSP70 have been reported to be associated with the heat tolerance, which provides clues to understand the mechanism of heat response. For example, the SNP 2437 T/C in the coding region of human HSPA1L is associated with heat shock response [15], and the −895 C insert-deletion (ins-del) in the promoter region of bovine HSP70.1 gene is associated with their summer heat tolerance by regulating the mRNA expression of HSP70.1 after heat stress [16]. In Chinese Holstein cattle, the haplotype 1 (TAATTACG) was associated with the heat-sensitive trait [17]. However, most of the studies on HSP70 polymorphisms were performed under laboratory conditions. As natural selections are usually followed by gene mutation [18], investigations on the genetic adaptations of closely related species in natural populations to different thermal conditions are of great interest [14]. Because of the limited studies on the polymorphisms of HSP70s in marine invertebrates, the molecular mechanisms of their contribution to differential heat tolerance in closely related populations is still far from well understood.

Bay scallop (*A. irradians*), first introduced from America in 1982, has become one of the most important economic bivalve species cultivated in China. However, in the past decades, the industry of scallop aquaculture had been suffering summer mortalities seriously, and the high temperature was suspected to be one of the main environmental inducer [19]. In order to prevent the tissue damage caused by heat stress, scallops have developed numerous self-protective strategies, such as the inducing synthesis of HSP70s. So far, one HSP70 gene has been identified (AiHSP70) from bay scallop [20], but there is no available information about the polymorphisms in its promoter and their associations with heat tolerance.

In the present study, two closely related populations of bay scallop inhabiting at different latitude were employed to investigate the molecular characters of HSP70 and their relationship with heat tolerance. One population distributed in Qinhuangdao, China (Northern Bay scallop, *A. i. irradians*) is a heat-sensitive species, which were introduced from Massachusetts of America in 1998, while the other population distributed in Zhanjiang, China (Southern Bay scallop, *A. i. concentricus*) is a more heat-resistant species, which were introduced from Florida of America in 1991 [21]. The objectives of the present study were to (1) examine the mRNA expression level of HSP70 genes in above two scallop populations, (2) screen the polymorphisms in the promoter region of HSP70, and analysis their association with the heat tolerance, (3) determine the *in vitro* promoter activity of HSP70 with different genotypes, and find a correlation between the population-specific molecular characteristics of the HSP70 gene promoter and the different adaptations of the examined populations to thermal stress.

## Materials and Methods

### Ethics statement

The bay scallops (averaging 45 mm in shell length) used in the present study were marine cultured animals, and were collected from a local farm in Qinhuangdao (119.57° E, 39.95° N) and Zhanjiang (110.38° E, 21.19° N) (China). No specific permits are required for the described field studies, since the bay scallops in the local farm are provided for the local market-sellings. And the bay scallop is not endangered or protected species. All the experiments were conducted according to the regulations of local and central government, and the study protocol was approved by the Experimental Animal Ethics Committee, Institute of Oceanology, Chinese Academy of Sciences, China.

### Scallop and acute heat stress treatment

Two hundred bay scallops, averaging approximately 45 mm in shell length, were collected from two scallop farms in Qinhuangdao (119.57° E, 39.95° N) and Zhanjiang (110.38° E, 21.19° N) (China), respectively, and the scallops from Zhanjiang are more heat-resistant than the scallops from Qinhuangdao [21]. The adductor muscle of each scallop from these two populations was collected and kept at −80°C until DNA isolation.

Another sixty scallops from the above two scallop farms were employed for the acute heat stress treatment experiment. After cultivation in 24 L tanks containing aerated seawater at 16°C for one week, 30 individuals of each population were averagely divided into five groups. The scallops in four groups were cultivated in 24 L tanks containing aerated seawater at 23°C, 27°C, 31°C and 35°C for two hours, respectively. And other 6 scallops were still kept in 24 L tanks containing aerated seawater at 16°C, and employed as the blank group. Gill from the scallops were collected and stored at −80°C with addition of 1 mL TRIzol reagent (Invitrogen) for subsequent RNA extraction.

### RNA isolation and cDNA synthesis

Total RNA was isolated from the gill of scallops using Trizol reagent (Invitrogen). The first strand cDNA synthesis was carried out based on manifacturer's instruction using the DNase I (Promega)-treated total RNA as template and oligo (dT)-adaptor primer P1 (Table 1). The reaction mixtures were incubated at 42°C for 1 h, and then terminated by heating at 95°C for 5 min. The cDNA mix was diluted to 1∶100 and stored at −80°C for subsequent SYBR Green fluorescent quantitative real-time PCR (RT-PCR).

**Table 1:** Primers used in this study.

Primer name	Sequence (5′-3′)	PCR objective
P1 (oligo (dT)-adaptor)	GGCCACGCGTCGACTAGTACT17	Clone primers
P2 (Ai70-RTF)	5′-GCGTAACACAACTGTCCCCAC-3′	RT-PCR
P3 (Ai70-RTR)	5′-TCATTGCTCGTTCTCCCTCG-3′	RT-PCR
P4 (AiActinF)	5′-CAAACAGCAGCCTCCTCGTCAT-3′	RT-PCR
P5 (AiActinR)	5′-CTGGGCACCTGAACCTTTCGTT-3′	RT-PCR
P6 (GSP1)	5′-GGTTGTCCTGTTTCCTTGGTCATTGGCG-3′	Genomic DNA Walking
P7 (AP1)	5′-GTAATACGACTCACTATAGGGC-3′	Genomic DNA Walking
P8 (GSP2)	5′-CGCCAGTTATTTCAATGTTCCTGTCTCA-3′	Genomic DNA Walking
P9 (AP2)	5′-ACTATAGGGCACGCGTGGT-3′	Genomic DNA Walking
P10 (proF)	5′-AAAACCATAACGGCTTGCCATACTAACC-3′	promoter cloning
P11 (proR)	5′-ATGCCACATAACTTGGGGTTGTCCTG-3′	promoter cloning
P12 (rproF)	5′-GGTACCATAACGGCTTGCCATACTAAC-3′	Recombination
P13 (rproR)	5′-AGATCTTTGCTAAAAACAAAAACGAAAT-3′	Recombination
P14 (RV-M)	5′-GAGCGGATAACAATTTCACACAGG-3′	Vector primer
P15 (M13-47)	5′-CGCCAGGGTTTTCCCAGTCACGAC-3′	Vector primer
P16 (RVp3)	5′-CTAGCAAAATAGGCTGTCCC-3′	Vector primer
P17 (GLp2)	5′-CTTTATGTTTTTGGCGTCTTCCA-3′	Vector primer

### DNA extraction and construction of genomic DNA walking library

The genomic DNA was extracted from the muscle of scallops using proteinase K and phenol/chloroform method [22]. The genomic DNA walking library was constructed using the GenomeWalkerTM Universal Kit protocol (Clontech). Following digestion with restrictive enzyme *Ssp* I, *Dra* I and *Pvu* II (NEB), the genomic DNA was purified by phenol/chloroform, and then ligated to GenomeWalkerTM adaptor [22].

### Real-time PCR analysis of AiHSP70 mRNA expression in two bay scallop populations

For SYBR Green fluorescent quantitative real-time PCR (RT-PCR), two AiHSP70-specific primers, sense primer P2 and reverse primer P3 (Table 1), were used to amplify the corresponding products. The scallop β-actin, amplified with primers P4 and P5 (Table 1), was chosen as reference gene for internal standardization. DEPC-water for the replacement of cDNA template was used as negative control.

The SYBR Green RT-PCR assay was carried out and analyzed as previously described [23]. The comparative average cycle threshold method was used to analyze the mRNA expression level of AiHSP70, and the value stood for an n-fold difference relative to the calibrator [24]. All data were given in terms of relative mRNA expressed as mean ± S.E. (N = 5). Differences were considered significant at *P*<0.05.

### Cloning and sequence analysis of AiHSP70 promoter

Two gene specific primers, P6 and P8 (Table 1) were designed based on the genomic DNA sequence of AiHSP70 to clone the promoter by genome walking approach [22]. The first round PCR amplification was performed by using gene specific primer P6 and adaptor primer P7 (Table 1). The nested PCR reaction was performed by using 1 µL of the first round PCR product (1∶50 dilution) as template and gene specific primer P8 and adaptor primer P9 (Table 1). All PCR amplification was performed in a PCR Thermal Cycle (TaKaRa, GRADIENT PCR). The PCR products were gel-purified and cloned into pMD18-T simple vector (TaKaRa, Japan). After being transformed into the competent cells of *E. coli* Top10, the positive recombinants were identified through anti-Amp selection and PCR screening with sense vector primer P14 and antisense vector primer P15 (Table 1). Three of the positive clones were sequenced on an ABI 3730 XL Automated Sequencer (Applied Biosystems).

The promoter sequence of AiHSP70 was analyzed using the Transcription Element Search System (TESS) (http://www.cbil.upenn.edu/cgi-bin/tess/tess) and the Patch System (http://www.gene-regulation.com/cgi-bin/pub/programs/patch/bin/patch.cgi?). The possible transcription start site was predicted using the Neural Network Promoter Prediction (NNPP) (http://www.fruitfly.org/seq_tools/promoter.html).

### Identification and analysis of polymorphisms in the promoter region of AiHSP70

A pair of gene specific primers, P10 and P11 (Table 1) was designed based on the promoter and coding sequence of AiHSP70 and used to amplify a 1680 bp fragment including the promoter region (1540 bp). The PCR products from six individuals from each of the two scallop populations were separated by electrophoresis on 1% agarose gels, respectively, and the fragments were excised and purified. The objective fragments were then cloned into pMD18-T vector (TaKaRa), transformed into *E. coli* Top10, and at least two clones were sequenced for each fragment using an ABI 3730 Automated Sequencer (Applied Biosystem). The alignments of nucleotide sequence of AiHSP70 promoter region were performed using Vector NTI Suite 9 and the polymorphisms in promoter region were identified from the sequence alignments of different individuals.

Based on the sites of polymorphisms identified by Vector NTI Suite 9, the amplified promoter region of AiHSP70 was analyzed by TFSEARCH program (http://www.cbrc.jp/research/db/TFSEARCH.html) to predict the putative transcription factor binding sites.

### Screening of polymorphisms in two scallop populations with differential heat tolerance

The 11 SNPs identified in the promoter region were screened by sequencing to examine their association with the heat tolerance of bay scallops. The genomic DNA (100 ng µL^−1^) of 50 individuals from each population was used as template to amplify the promoter region with gene specific primers P10 and P11 (Table 1). The PCR products were gel-purified and sequenced with gene specific primers P10 and P11 (Table 1).

The genotype data at the 11 tested SNP sites was analyzed to test Hardy-Weinberg equilibrium (HWE). Moreover, linkage disequilibrium (LD) test and haplotype analysis were also conducted according to the genotyping results. All the data were analyzed by using SHEsis software (http://analysis.bio-x.cn) [25]. A *P*-value less than 0.05 was accepted as significant.

### Dual luciferase reporter assays of *AiHSP70* promoter with different genotypes

To investigate the luciferase reporter activity of *AiHSP70* promoter with heat sensitive genotype (G-C-A-A-C-T-C-A-C-A-A) (pHSP70S) and heat resistant genotype (A-A-T-C-G-A-A-T-G-G-G) (pHSP70R), two target *AiHSP70* promoter-reporter plasmids were constructed (pGL3-Basic-pHSP70S and pGL3-Basic-pHSP70R). The DNA fragments, which encompassed −1382 to −1 bp (relative to the transcription start site) of *AiHSP70*-promoter region, were amplified with gene specific primers P12 and P13 (Table 1) from individuals with different genotype, respectively. A *Kpn* I site and a *Bgl* II site was added to the 5′ end of sense primer P12 and antisense primer P13 (Table 1), respectively. The PCR fragments were digested completely by restriction enzymes *Kpn* I and *Bgl* II (NEB), and then cloned into the *Kpn* I/*Bgl* II sites of pGL3-Basic vector (Promega, Madison, WI, USA) containing the firefly luciferase gene as a reporter. The positive constructs were identified through PCR screening with sense vector primer P16 and antisense vector primer P17 (Table 1), confirmed by DNA sequencing, and finally extracted by endotoxin-free plasmid extraction kit (Promega).

Hela cells were seeded in 24-well culture plates (1–2×10^5^ cells/well) 24 hours prior to transfection, and cultured in RPMII 1640 media (Hyclone) supplemented with 10% fetal bovine serum (FBS, Invitrogen) at 5% CO_2_, 37°C. Cells (85–95% confluence) were transfected with 0.8 µg of each *AiHSP70*-promoter reporter plasmid (pGL3-Basic-pHSP70S and pGL3-Basic-pHSP70R) and 0.08 ng of pRL-TK plasmid (Promega) as a normalizing control by using Lipofectamine 2000 (Invitrogen, Carlsbad, CA, USA) according to the manufacturer's instructions. The pGL3-Basic and pGL3-Control vector were also transfected as negative and positive control, respectively. After 24 hours' transfection, cells were treated at 45°C for 1 h and cultured at 5% CO_2_, 37°C for another 24 hours. The cells without heat treatment were collected as blank control.

The cells were lysed after 48 hours transfections with the passive lysis buffer (Promega) for luciferase assays. Firefly and Renilla luciferase activities were measured using the Dual-Luciferase Reporter Assay System (Promega), and the promoter activity was expressed by Relative Luciferase Activity (RLA). Three independent experiments were performed with triplicate wells. Differences were considered significant at *P*<0.05.

## Results

### Basal and heat-induced temporal expression of AiHSP70 mRNA in two bay scallop populations

**Figure 1:**
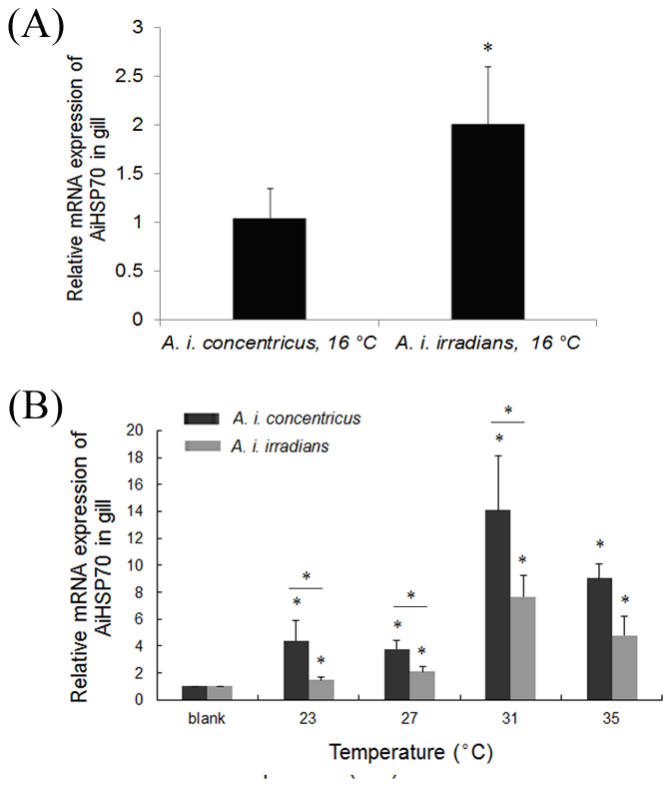
Temporal expression of AiHSP70 mRNA in bay scallop *A. i irradians *and *A. i. concentricus* relative to β-actin in gills under normal physiological condition (A) and after acute heat stress treatment (B) as measured by RT-PCR. Vertical bars represent the mean ± S.E. (N = 5). (*: *P*<0.05).

The basal expression level of HSP70 mRNA in the heat resistant *A. i. concentricus* under normal physiological conditions (16°C) was 2.01-fold of that in the heat sensitive *A. i. irradians* (*P*<0.05) (Fig. 1A). The relative expression levels of HSP70 mRNA were significantly up-regulated in both heat resistant *A. i. concentricus* and heat sensitive *A. i. irradians* after acute heat stress treatments in a temperature-dependent manner (*P*<0.05), and reached the maximum levels at 2 h after the treatment of 31°C. A significant drop of HSP70 synthesis was observed in scallops of both stocks after the treatment with 35°C (Fig. 1B). After 2 h of acute heat stress treatment at 23°C, 27°C, 31°C and 35°C, the mRNA levels of HSP70 in *A. i. concentricus* and *A. i. irradians* were 4.36-fold (*P*<0.05), 3.77-fold (*P*<0.05), 14.09-fold (*P*<0.05) and 9.02-fold (*P*<0.05), and 1.47-fold (*P*<0.05), 2.12-fold (*P*<0.05), 7.68-fold (*P*<0.05) and 4.79-fold (*P*<0.05) of that in the blank, respectively. There were significant differences between the mRNA levels of HSP70 in *A. i. concentricus* and *A. i. irradians* after the heat stress treatments at 23°C, 27°C and 31°C (*P*<0.05) (Fig. 1B).

### The sequence features of AiHSP70 promoter

**Figure 2:**
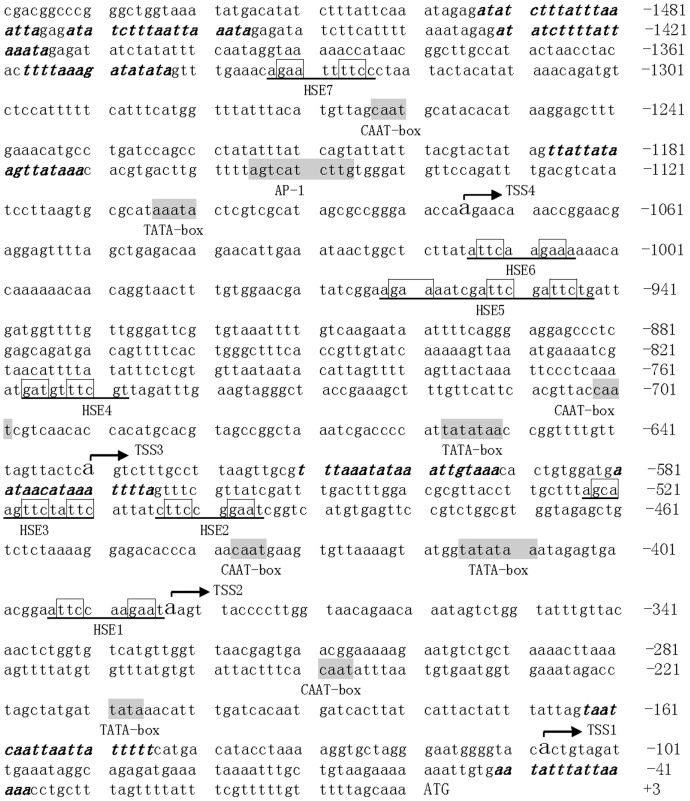
Sequence analysis of AiHSP70 promoter. The negative numbers on the right indicate upstream sequence relative to the translation start codon and the putative promoter elements are marked. The putative transcription start sites (TSS) are shown in bigger letters and curved arrow. The TATA box, CAAT box and one cis-element (AP-1) are shadowed by gray. The AT-rich regions are indicated by bold italic. The HSEs are indicated with underline and the core sequences (GAA/TTC) are boxed.

The promoter region of AiHSP70 (1540 bp) was amplified by genomic DNA walking approach and the promoter sequence was presented in Fig. 2. The A, T, G, and C base content of AiHSP70 promoter region was 33.05%, 33.9%, 17.66% and 15.39%, respectively, indicating that the AiHSP70 promoter region was an AT rich region (66.95%). Four possible transcription start sites (TSS1-TSS4) located at −109 bp, −384 bp, −631 bp and −1076 bp upstream of the ATG codon were identified in AiHSP70 promoter based on the NNPP database (Fig. 2 and Table S1).

There were several core promoter elements in AiHSP70 promoter, including four TATA boxes located at −207 bp, −410 bp, −652 bp and −1001 bp upstream of the ATG codon, and four CAAT boxes located at −247 bp, −435 bp, −700 and −1261 bp upstream of the ATG codon (Fig. 2). With the help of NNPP, TESS and Patch databases, several putative cis-acting elements were found in the AiHSP70 promoter, such as one activating protein-1 (AP-1) and seven classical heat shock elements (HSE1-7) with the core consensus sequence 5′-nGAAn-3′ [26]. The nearest HSE1 (aTTCcaaGAAt) and the farthest HSE7 (aGAAttTTCc) located at −385 bp and −1325 bp upstream of the ATG codon, respectively (Fig. 2). The other five HSEs, including HSE2 (cTTCcgGAAt), HSE3 (aGCAagTTCtaTTCa), HSE4 (tGATgtTTCg), HSE5 (aGAAaatcgaTTCgaTTCt) and HSE6 (aTTCaaGAAa) located between −496 bp and −1015 bp upstream of the ATG codon (Fig. 2). These HSEs might be involved in the regulation of heat shock response by interacting with heat shock factors (HSFs).

### The polymorphisms in the promoter region of AiHSP70

**Figure 3:**
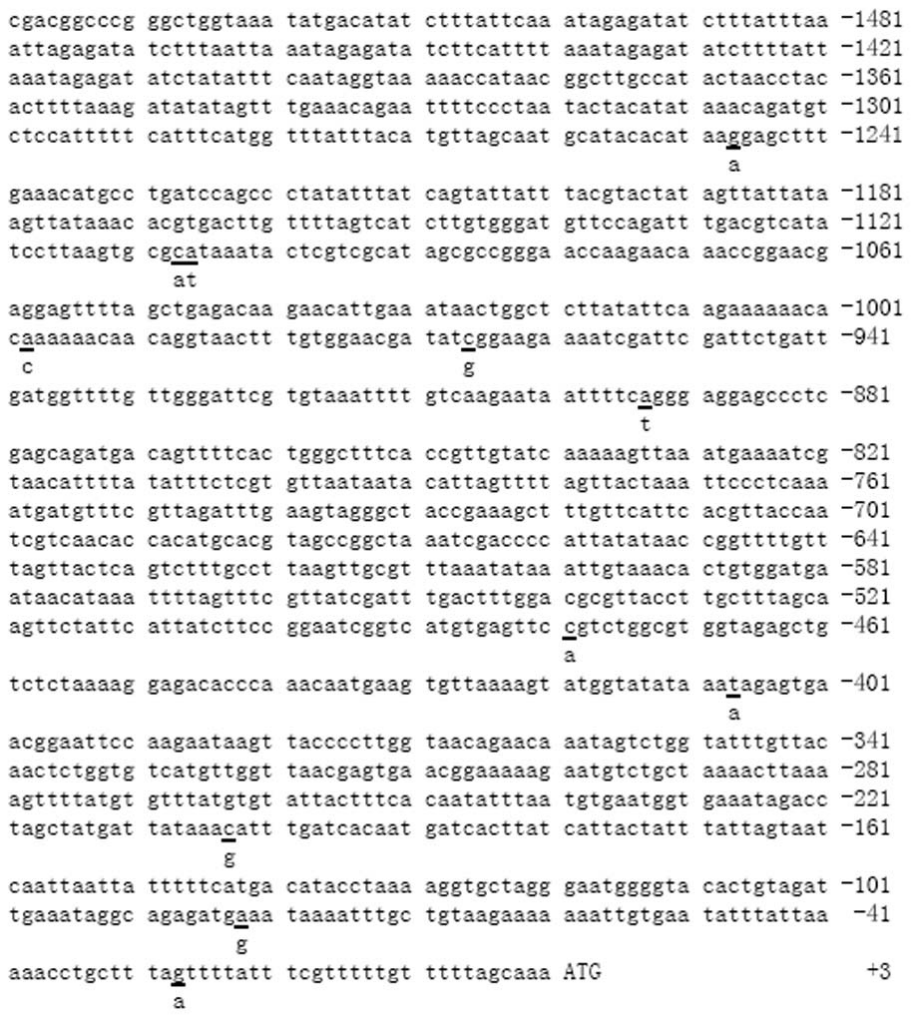
The polymorphisms in the promoter region of AiHSP70. The negative numbers indicate upstream sequence relative to the translation start codon and are numbered on the right. The polymorphism sites are underlined and the variants are described below.

A 1540 bp fragment in AiHSP70 promoter region was amplified from bay scallops, and 18 positive clones from six scallops from each of heat resistant and sensitive populations were sequenced. Eleven SNPs, including −1248 G-A, −1108 C-A, −1107 A-T, −999 A-C, −967 C-G, −894 A-T, −480 C-A, −408 T-A, −204 C-G, −83 A-G and −28 G-A were found in the amplified promoter region, and two of them (−1108 and −1107) were in close linkage (Fig. 3).

**Figure 4:**
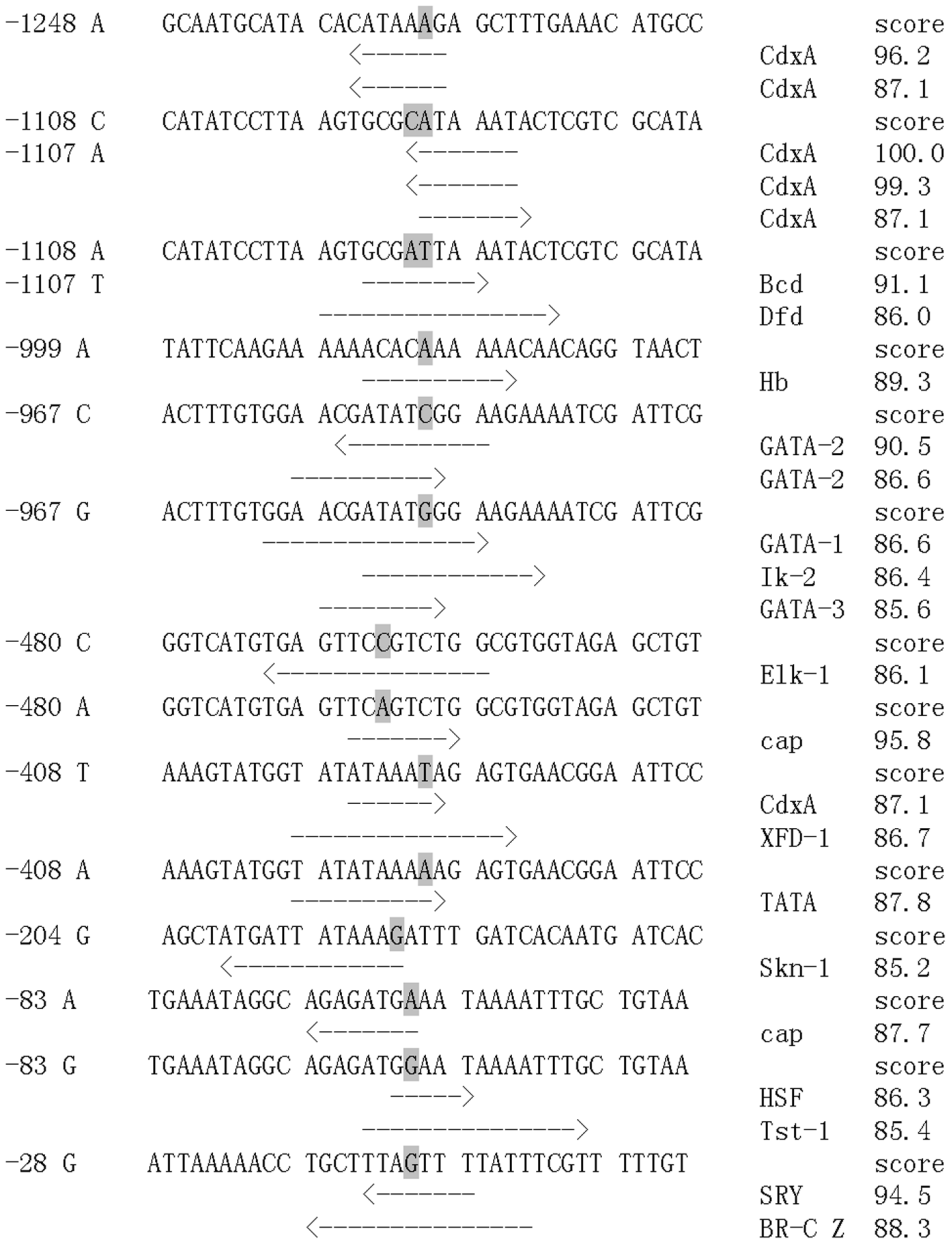
Difference of putative binding sites for transcription factors between different alleles. Polymorphic loci are shadowed in gray and the first polymorphic locus of each line is numbered on the left. The positions and directions of putative binding sites for transcription factors are labeled by arrowhead. The TFSEARCH scores are shown on the right.

The putative transcription factor binding sites were screened at these 11 sites by TFSEARCH analysis and their difference between two alleles was marked in Fig. 4. At four loci (−1248, −999, −204 and −28), there were some more putative transcription factor binding sites in one allele than their corresponding allele. For example, there were two more putative CdxA binding sites in −1248 A allele than G allele, one more putative Hb binding site in −999 A allele than C allele, and one more putative Skn-1 binding site in −204 G allele than C allele, respectively. At locus −28, there was one more putative binding site for SRY and BR-C Z in G allele than A allele. At the other 7 loci, the polymorphisms changed the respective transcription factor binding sites in each allele, including locus −1108, −1107, −967, −894, −480, −408 and −83. For example, at the loci −1108 and −1107 which were in close linkage, three more putative CdxA binding sites were found in CA allele, while one more putative binding site for Bcd and Dfd were found in AT allele. At locus −83, one more putative Cap binding site was found in A allele instead of one more putative binding sites for HSF and Tst-1 in G allele. The nucleotides are numbered on the left and the polymorphic sites are underlined (Fig. 4).

### The association between SNPs in AiHSP70 gene and heat tolerance of bay scallops

**Figure 5:**
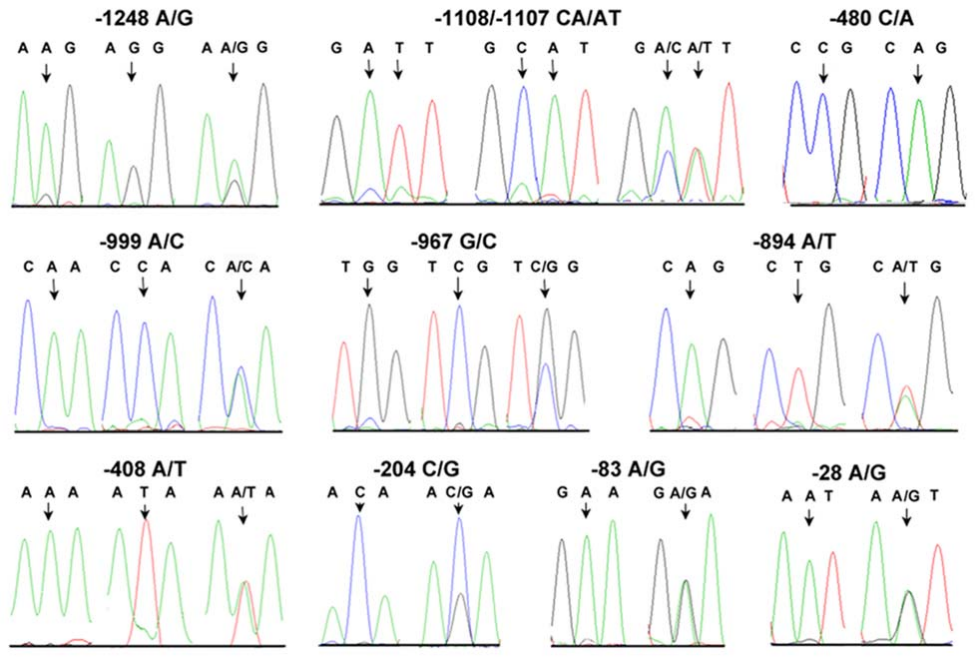
Detection of the genotypes at 11 SNPs in the AiHSP70 promoter region. The unimodal peak indicates the homozygous genotype, and the overlapping peaks indicate the heterozygous genotype.

The association between the sequence polymorphism and heat tolerance was investigated by examining frequency distribution of all the 11 promoter polymorphic loci in the heat sensitive and heat resistant populations. In the sequencing map, homozygous genotype had unimodal peak, while that of heterozygous genotype had overlapping peaks (Fig. 5). For example, the sequencing map of −1248 AA and −1248 GG genotypes displayed their specific unimodal peak of A or G, respectively, while the sequencing map of −1248 AG genotype displayed overlapping peak of both A and G. Seven of the 11 polymorphic loci (locus −1248, −1108, −1107, −999, −967, −894 and −408) had three genotypes reflected by three kinds of sequencing maps, while the other four loci (locus −480, −204, −83 and −28) had two genotypes reflected by two kinds of sequencing maps.

The allele and genotype frequencies at 11 loci in the AiHSP70 promoter region were summarized in Table 2. The Hardy-Weinberg equilibrium for genotype frequencies was analyzed with the goodness-of-fit χ^2^-test. Statistical analysis revealed that the genotype frequency of alleles at six loci (locus −1248, −1108, −1107, −999, −894 and −204) were in HWE (*P*>0.05) in both populations, while that at locus −28 was not in HWE (*P*<0.05) in both populations (Table 2). The genotype frequency of alleles at the other three loci (locus −967, −480 and −83) were in HWE (*P*>0.05) in the heat sensitve population, while not in HWE (*P*<0.05) in the heat resistant population, and that of the locus −408 was in contrary to those of the above three loci (Table 2). The allele and genotype frequencies at 8 loci (−1248, −1108, −1107, −999, −967, −480, −408 and −83) were significant different in two populations (*P*<0.05), while no significant difference was observed at locus −28 in two populations (*P*>0.05) (Table 2). As for locus −894, the allele frequency exhibited significant difference in two populations (*P*<0.05), while no significant difference was found for its three genotypes in two populations (*P*>0.05). In contrast, allele frequency of locus −204 exhibited no difference in two populations (*P*>0.05), while the frequencies of its two genotypes were significantly different in two populations (*P*<0.05) (Table 2).

**Table 2:** Allele and genotype frequencies of different SNPs in *A. i. irradians* and *A. i. concentricus* populations.

SNP		*A. i. irradians* No. (%)	*A. i. concentricus* No. (%)	?^2^ (*P*)
−1248 G/A	Allele G	60 (96.8)	29 (46.8)	38.25 (*P*<0.01)
	A	2 (3.2)	33 (53.2)	
	Genotype G/G	29 (93.5)	8 (25.8)	29.98 (*P*<0.01)
	G/A	2 (6.5)	13 (41.9)	
	A/A	0 (0)	10 (32.3)	
	HWE test Pearson χ2 (*P*)	0.034 (0.853)	0.77 (0.379)	
−1108 C/A	Allele C	55 (88.7)	34 (54.8)	17.56 (*P*<0.01)
	A	7 (11.3)	28 (45.2)	
	Genotype C/C	24 (77.4)	7 (22.6)	19.58 (*P*<0.01)
	C/A	7 (22.6)	20 (64.5)	
	A/A	0 (0.00)	4 (12.9)	
	HWE test Pearson χ2 (*P*)	0.50 (0.478)	2.84 (0.092)	
−1107 A/T	Allele A	55 (88.7)	34 (54.8)	17.56 (*P*<0.01)
	T	7 (11.3)	28 (45.2)	
	Genotype A/A	24 (77.4)	7 (22.6)	19.58 (*P*<0.01)
	A/T	7 (22.6)	20 (64.5)	
	T/T	0 (0.00)	4 (12.9)	
	HWE test Pearson χ2 (*P*)	0.50 (0.478)	2.84 (0.092)	
−999 A/C	Allele A	55 (88.7)	31 (50.00)	21.86 (*P*<0.01)
	C	7 (11.3)	31 (50.00)	
	Genotype A/A	24 (77.4)	6 (19.4)	22.34 (*P*<0.01)
	A/C	7 (22.6)	19 (61.3)	
	C/C	0 (0)	6 (19.4)	
	HWE test Pearson χ2 (*P*)	0.50 (0.479)	1.58 (0.208)	
−967 G/C	Allele G	5 (8.1)	34 (54.8)	31.46 (*P*<0.01)
	C	57 (91.9)	28 (45.2)	
	Genotype G/G	0 (0)	6 (19.4)	32 (*P*<0.01)
	C/G	5 (16.1)	22 (71)	
	C/C	26 (83.9)	3 (9.7)	
	HWE test Pearson χ2 (*P*)	0.24 (0.625)	5.81 (0.016)	
−894 A/T	Allele A	17 (27.4)	29 (46.8)	4.98 (*P*<0.05)
	T	45 (72.6)	33 (53.2)	
	Genotype A/A	2 (6.5)	8 (25.8)	4.98 (*P*>0.05)
	A/T	13 (41.9)	13 (41.9)	
	T/T	16 (51.6)	10 (32.3)	
	HWE test Pearson χ2 (*P*)	0.089 (0.765)	0.77 (0.379)	
−480 A/C	Allele A	0 (0)	44 (71)	68.2 (*P*<0.01)
	C	62 (100)	18 (29)	
	Genotype A/A	0 (0)	22 (71)	34.1 (*P*<0.01)
	C/C	31 (100)	9 (29)	
	HWE test Pearson χ2 (*P*)	0.00 (1.000)	31 (2.64e-008)	
−408 A/T	Allele A	35 (56.5)	27 (43.5)	2.06 (*P*<0.05)
	T	27 (43.5)	35 (56.5)	
	Genotype A/A	5 (16.1)	4 (12.9)	6.37 (*P*<0.05)
	A/T	25 (80.6)	19 (61.3)	
	T/T	1 (3.2)	8 (25.8)	
	HWE test Pearson χ2 (*P*)	12.71 (0.000)	1.88 (0.169)	
−204 C/G	Allele C	58 (93.5)	51 (82.3)	3.72 (*P*>0.05)
	G	4 (6.5)	11 (17.7)	
	Genotype C/C	27 (87.1)	20 (64.5)	4.31 (*P*<0.05)
	C/G	4 (12.9)	11 (35.5)	
	HWE test Pearson χ2 (*P*)	0.15 (0.701)	1.44 (0.229)	
−83 A/G	Allele A	58 (93.5)	34 (54.8)	24.26 (*P*<0.01)
	G	4 (6.5)	28 (45.2)	
	Genotype A/A	27 (87.1)	4 (12.9)	34.13 (*P*<0.01)
	A/G	4 (12.9)	27 (87.1)	
	HWE test Pearson χ2 (*P*)	0.147 (0.701)	18.44 (1.77e-005)	
−28 A/G	Allele A	38 (61.3)	36 (58.1)	0.134 (*P*>0.05)
	G	24 (38.7)	26 (41.9)	
	Genotype A/A	7 (22.6)	6 (19.4)	0.097 (*P*>0.05)
	A/G	24 (77.4)	25 (80.6)	
	HWE test Pearson χ2 (*P*)	12.37 (0.0004)	14.15 (0.0002)	

The frequencies of individuals with genotype −1248 GG, −1108 CC, −1107 AA, −999 AA, −967 CC, −480 CC, −408 AA and −83 AA in the heat sensitive population were significantly higher than that in the heat resistant population (*P*<0.05), while the frequencies of individuals with genotype −1248 AA, −1108 AA, −1107 TT, −999 CC, −967 GG, −480 AA, −408 TT and −83 AG in the heat resistant population were significantly higher than that in the heat sensitive population (*P*<0.05), and scallops with genotype −480 AA were only identified in the heat resistant population (Table 2). Therefore, the eight genotypes (−1248 AA, −1108 AA, −1107 TT, −999 CC, −967 GG, −480 AA, −408 TT and −83 AG) of AiHSP70 were considered as gene markers associated with heat tolerance.

**Figure 6:**
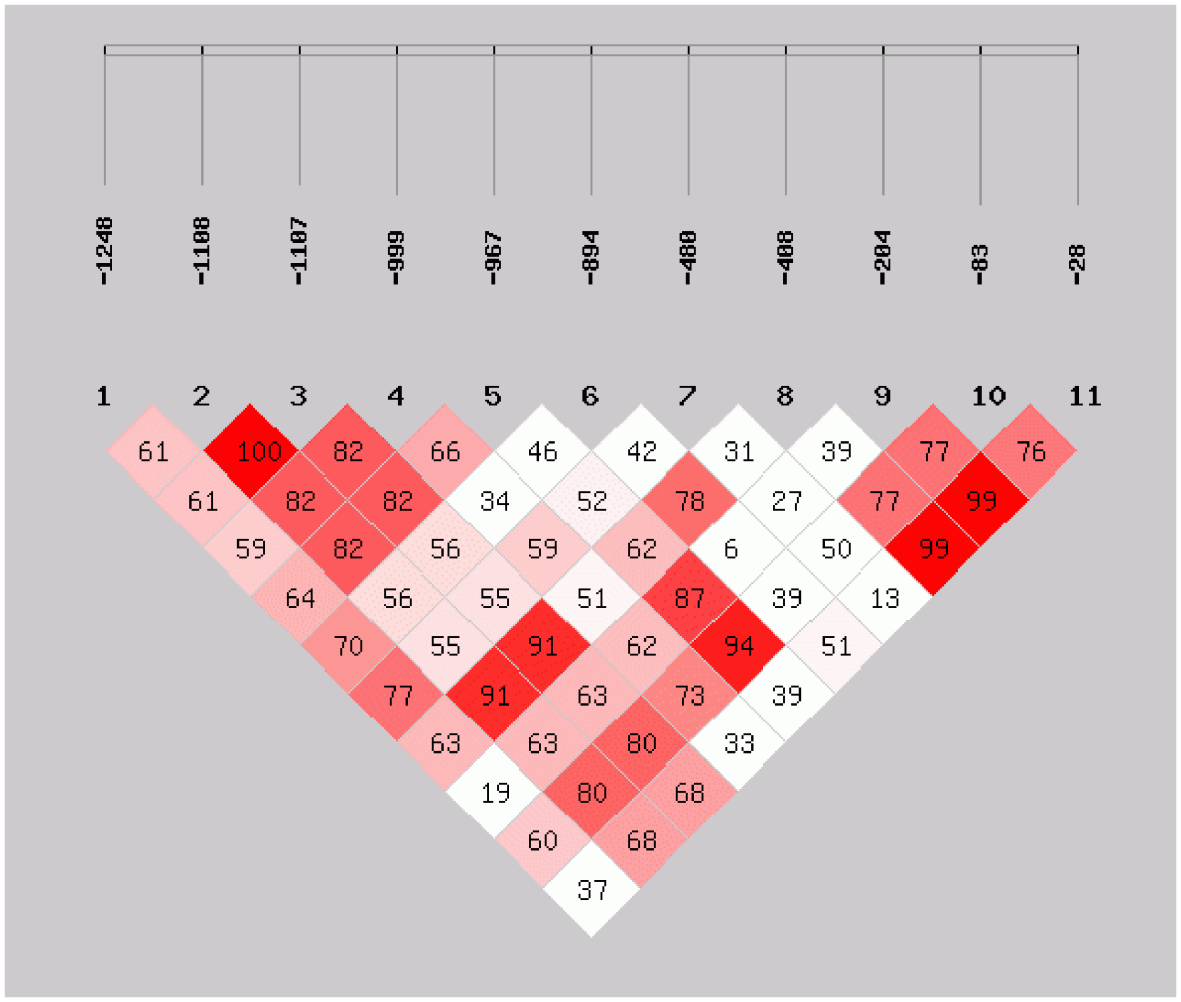
The linkage disequilibrium analysis of the 11 SNPs in the promoter region of AiHSP70. SHEsis software (http://analysis2.bio-x.cn/myAnalysis.php) was used for this analysis. The colour scheme is: white (D = 0), pink (0<D<1), red (D = 1).

D' value is one of the important parameters to measure the LD between two loci, and the LD between two loci can be divided into three cases according to their D' values, including completely independent (D' = 0), complete LD (D' = 1) and a certain degree of LD (0<D'<1). The result of the pair-loci LD test revealed that the loci in AiHSP70 gene promoter were in different degree of LD. Four pair loci (−1108 and −1107, −967 and −204, −967 and −83, −408 and −28) were in strong LD with a pairwise D'>0.85 (Fig. 6, Table S2). Considering that the LD between different polymorphic loci in one gene usually form different haplotypes, two pair of loci (−1108 and −1107, −408 and −28), which were in complete LD, were selected for haplotype analysis. These 4 polymorphic loci could form 6 haplotypes with frequency greater than 0.01, and the frequency of haplotype ATTG in heat resistant population was significantly higher than that in heat sensitive population (OR = 5.845, 95% CI 2.182–15.658, *P*<0.01), while the frequency of haplotype CATG in heat sensitive population was significantly higher than that in heat resistant population (OR = 0.045, 95% CI 0.007–0.316, *P*<0.01) (Table S3).

### The transcriptional activity of AiHSP70 gene promoters with different genotypes

**Figure 7:**
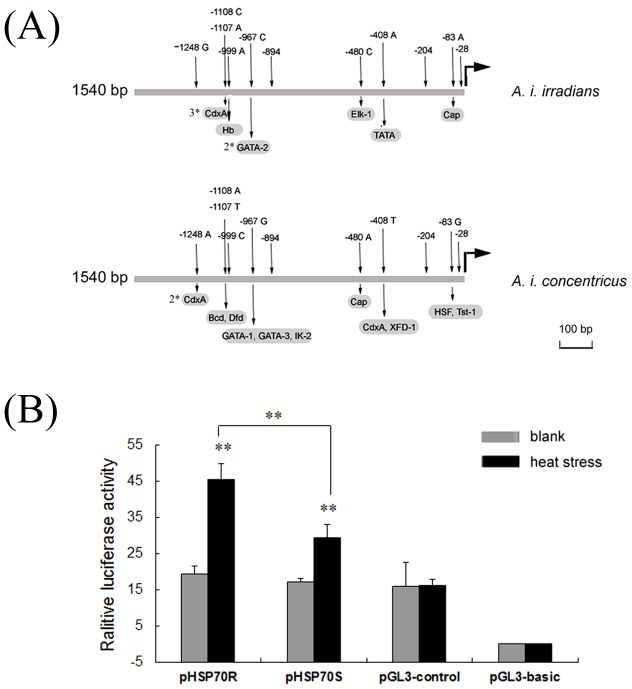
Dual luciferase report assay of AiHSP70 promoter with different haplotypes. (A) The dominant alleles and their corresponding putative binding sites for transcription factors in the HSP70 promoter-reporter constructs of *A. i. irradians* and *A. i. concentricus*. The Arabic numerals before the name of transcription factors indicate their amount; the black bent arrows mark the positions of the start codon, ATG, and the direction of transcription. (B) Relative luciferase activity (RLA) of Firefly and Renilla luciferase driven by AiHSP70 promoter. Two constructs containing the AiHSP70 promoter with thermosensitive haplotype (pHSP70S) and thermoresistant haplotype (pHSP70R) were solely transfected into Hela cells while the pGL3-basic transfected cells served as negative control (pGL3-basic) and the pGL3-control transfected cells served as positive control (pGL3-control). The pRL-TK Renilla luciferase vector was used as an internal control, and RLA of Firefly and Renilla luciferase was calculated. Vertical bars represent the mean ± S.E. (N = 6). (**: *P*<0.01).

To test the effects of promoter polymorphisms on HSP70 transcription *in vitro*, two luciferase reporter plasmids with the dominant alleles of *A. i. irradians* and *A. i. concentricus* were constructed, respectively (Fig. 7A). There were two more putative GATA-2 binding sites and one more putative Hb, TATA and Elk-1 binding sites in the AiHSP70 promoter from *A. i. irradians* with alleles G-C-A-A-C-T-C-A-C-A-A, while there was one more putative binding sites for transcription factor Bcd, Dfd, GATA-1, GATA-3, Ik-2, XFD-1, HSF and Tst-1 in the AiHSP70 promoter from *A. i. concentricus* with alleles A-A-T-C-G-A-A-T-G-G-G (Fig. 7A).

Compared to the blank group, the transcriptional activities of AiHSP70 gene promoter (pHSP70R and pHSP70S) increased by 2.34-fold (*P*<0.01) and 1.73-fold (*P*<0.01) after heat stress treatment at 45°C for 1 h, respectively (Fig. 7B). And the transcriptional activity of pHSP70R was significantly higher than that of pHSP70S (1.54-fold, *P*<0.01) in the heat stress group, while no significant difference was found between them in the blank group (Fig. 7B). The RLA of Hela cells containing pGL3-basic vector was less than 0.05 in both blank and heat stress group, while that of Hela cells containing pGL3-control vector was 15.89 in the blank group and 15.99 in the heat stress group, respectively (Fig. 7B).

## Discussion

Though the fluctuation of HSP levels is not ‘a silver bullet’ and organisms may exhibit different strategies for adaptation to adverse environmental conditions [14], [27], stress-induced synthesis of HSPs represents a generalized molecular mechanism for almost all cells [28], and the induction of HSP70 synthesis by heat shock and other stressors has previously been reported in various mollusc species [29]. Moreover, the expression timing and/or intensity of HSP70 were found to be different in thermally adapted species of marine mollusc in response to acute thermal stress [30], [31]. In the present study, the relative mRNA expressions of HSP70 in two bay scallop populations were investigated under normal physiological condition and after acute heat stress treatment. The heat resistant scallop *A. i. concentricus* inhabiting approximately 21° N (subtropical climate, the temperature of sea water could reach 31°C in summer) displayed a lower basal level and a stronger induction of HSP70 mRNA in response to the acute heat stress treatment, compared with the heat sensitive scallop *A. i. irradians* from approximately 40° N (warm temperate climate, the temperature of sea water was generally below 28°C in summer). It is worth mentioning that other factors can also affect the heat resistance of the species. But considering the living environment of these two scallops in the United States and China, we believe that different latitudinal distribution is the main factor among all the living environment factors, while different latitudinal distribution is directly related with temperature tolerance. In consideration of the important roles of HSP70 in modulating heat stress response, it was speculated that the high basal level of HSP70 allowed *A. i. irradians* to adapt the low temperature in winter and the high inducing level of HSP70 allowed *A. i. concentricus* to overcome the stress of high temperature in summer. And the regulation of HSP70 expression may be regarded as one of the adaptation mechanisms of scallop evolved to drastic temperature fluctuations. Similar result was also reported in a cold-adapted species *Mytilus trossulus* which displayed higher constitutive expression of HSP70 and lesser ability to induce this protein compared to a warm-adapted species *M. galloprovincialis* [32]. However, there were also some reports with inconsistent results. Lockwood et al. reported that the cold-adapted mussel species *M. trossulus* and the warm-adapted species *M. galloprovincialis* exhibited similar timing and intensity of expression of HSP70 gene [33]. In Lake Baikal amphipods, the heat resistant species *Eulimnogammarus cyaneus* displayed a higher basal level of HSP70 mRNA and a less pronounced HSP induction in response to heat stress compared with the heat sensitive *E. verrucosus*, and the high constitutive level of HSP70 was suggested to represent a general protective mechanism against heat stress [14]. Whole-transcriptome or transcriptome sequencing approach may be powerful biology research tools to explain these inhomogenous HSP70 expression trends. Though there was some inconsistence in the previous studies, the regulation of the HSP70 gene expression was believed to play important roles in heat stress response.

The expression of inducible HSP70 gene is mainly regulated at transcriptional level depending on the interaction between transcription factor and their putative cis-acting elements in the promoter region of HSP70 genes [22], [34]. Although the promoters of HSP70s and their response to environmental stress have been well studied in vertebrates [35], [36], the corresponding information in invertebrates especially in mollusc is still limited [29], [37]. By now, the promoter elements of HSP70s were only predicted in a few seawater species. The available information of the few homologous HSP70s was not enough to create a figure which could help to compare the promoter elements of homologous HSP70s. In the present study, a fragment of 1540 bp in the promoter region of AiHSP70 was amplified from bay scallop, and some putative cis-acting elements HSEs were identified, implying the structural basis of AiHSP70 for heat stress response. In mollusc, it has been reported that the promoter of HSP70 gene could be activated by heat stress [10], [38], [39]. The heat induced expression pattern of AiHSP70 mRNA was consistent with the structure of AiHSP70 promoter.

Since stress-induced synthesis of HSPs represents a generalized molecular mechanism, the differences in stress adaption of individual animals may be partly attributed to the existence of polymorphisms in a series of related genes [28]. It has been reported that the polymorphisms occurring naturally in the functional promoter regions affect the expression of HSP70 and contribute to differential stress tolerance [16], [28], [34]. In the present study, a total of 11 SNP sites were found in the promoter region of AiHSP70, which changed the binding sites for certain transcription factors, and they might affect the transcriptional efficiency. This speculation could partly explain the different expression pattern of AiHSP70 mRNA under heat stress in the two scallop species, and the similar results have also been reported in bovine, which showed one cytosine ins-del polymorphism in AP2 box region of HSP70.1 gene affected the mRNA expression of HSP70.1 after heat stress, and finally displayed different thermal stress response [16].

Considering SNPs in the promoter region of AiHSP70 gene might be the cause of different expression pattern of AiHSP70 mRNA under heat stress in two species, association analysis between these SNPs and heat tolerance of bay scallop were performed based on case-control studies [40]. Statistical analysis revealed that the genotype frequency of alleles at six loci (locus −1248, −1108, −1107, −999, −894 and −204) were in HWE (*P*>0.05) in both populations, while that at the other five loci (locus −967, −480, −408, −83 and −28) was not in HWE (*P*<0.05) in at least one stock. As mutational events usually followed by environmental selection [18], which could be reflected by deviation from HWE [41], the disequilibrium of the genotype frequency at locus −967, −480, −408, −83 and −28 might imply a genetic adaptation process. In order to verify this extrapolation, the allele and genotype frequencies at 11 loci in the AiHSP70 promoter region were calculated and compared. The allele and genotype frequencies at 8 loci (−1248, −1108, −1107, −999, −967, −480, −408 and −83) were significantly different in the two populations (*P*<0.05). Together with the HWE analysis results, a total of four SNP sites (−967, −480, −408 and −83) could be considered as the heat sensitivity/tolerance related SNP candidates. A case-control study is an analytical study in which two existing groups differing in outcome are identified and compared on the basis of some supposed causal attribute, which has been widely used for association analysis between DNA sequence variants and various traits, and also applied to identify risk-enhancing or protective genotypes [16], [17], [22], [42]. As the −967 GG, −480 AA, −408 TT and −83 AG genotypes were more prevalent in the heat resistant population than that in the heat sensitive population, they were suggested to be significantly associated with the resistant phenotype of bay scallops to heat stress and could be potential markers in the selection of scallops with heat resistant traits. The characterization of such mutation has important implications for understanding of the genetic adaptation processes in organisms [18].

As most of the resistance traits are of quantitative and they are always controlled by multiple genes [43], the effect of a single SNP might be a small part of the causes for the related traits. In the present study, the comprehensive LD and haplotype analyses of SNPs in AiHSP70 gene promoter were performed to examine the possible interactions between the genotypes and the heat tolerance. The pair-loci LD test revealed that the 11 SNPs were in different degree of LD, and four pair loci (−1108 and −1107, −967 and −204, −967 and −83, −408 and −28) were in strong LD with a pairwise D'>0.85. Because the LD between different polymorphic loci in one gene usually leads to the formation of different haplotypes, two pair loci in complete LD (−1108 and −1107, −408 and −28) were selected for haplotype analysis, and statistical analysis showed that they could form six haplotypes. The frequency of haplotype ATTG in heat resistant population was significantly higher than that in heat sensitive population, therefore the haplotype ATTG was believed to confer better heat tolerance to the scallops compared with that of other haplotypes. However, the heat resistant haplotype candidate only included one heat tolerance related SNP according to the combined results of HWE and allele frequencies analysis. It seemed that the result of haplotype analysis was inconsistent with the combined results of HWE and allele frequencies analysis. In fact, the population genetics studies about the balancing selection have confirmed that genetic adaptation not only maintains diversity at the selected sites but increases diversity at closely linked neutral sites [17]. The knowledge also provides an explanation for the different number of heat-resistant haplotype candidate and SNP candidates in the present study.

Recently, there is a growing interest in investigating the effect of genetic variations in HSP70 on heat tolerance [15]–[17]. Unfortunately, most of the reports are limited to association analysis without experimental evidences. In the present study, Hela cells were used in luciferase assay and the association between polymorphisms and heat tolerance were subsequently confirmed by further functional analysis of the AiHSP70 promoter with two different genotypes. It has been reported that mammal cell lines can be used in invertebrates' studies [44], [45]. The results showed that the RLA of Hela cells containing pGL3-basic vectors was very low while that containing pGL3-control vector was unchanged, indicating that the background signal of pGL3 vector could be ignored and the transient transfection system was relatively stable during the entire experiment. In luciferase assay no significant difference was found in the blank activity while there are differences in previous natural experiments (Figure 1). The reason maybe that luciferase assay is a strictly controlled and credible experiment which is influenced by only one factor. But for in vivo experiments, the gene expression may be affected by multiple factors, and the expression of HSP70 may be associated with other genes. After 1 h heat shock at 45°C, the transcriptional activity of pHSP70R was significantly higher than that of pHSP70S, which was in accordance with the different temporal expression pattern of AiHSP70 mRNA in two bay scallop populations after acute heat stress treatment. TFSEARCH analysis also revealed that there were some differences in the putative transcriptional factor binding sites among the 11 SNPs, and it was noteworthy that −83 G allele had one more putative HSF binding site than A allele. The polymorphisms in the promoter region in genes of other organisms have also been reported to affect the gene expression by changing putative binding sites for some key transcriptional factor [16], [31], [46]. As the cDNA sequence of HSF in bay scallop has not been identified, whether or not the SNP −83 A-G will affect the interaction between HSF and the promoter of AiHSP70 is unknown at the present. The functional analysis in the present study drew a biologically rational mechanism of the association between promoter polymorphism and mRNA expression, and the SNP −83 A-G in the AiHSP70 promoter was believed to have a causal effect on the different heat tolerance of two scallop populations.

In summary, the mRNA expression patterns of AiHSP70 gene in two bay scallop populations under normal physiological and heat stress conditions were found to be different, and the *in vitro* promoter activities of AiHSP70 with different genotypes after heat stress treatments were also different significantly. Significant association between the promoter polymorphisms and heat tolerance was established in bay scallop, and some potential gene markers associated with enhanced heat tolerance could be applied in the future molecular assisted selection program of bay scallop. The present results indicated that the peculiarities of HSP70 gene expression were closely coupled with the adaptation to the fluctuating thermal habitats for scallops.

## Supporting Information

Table S1 **The potential transcription start sites in the promoter of AiHSP70 gene.**
(DOCX)

Table S2 **The D' value between the polymorphic loci in the promoter region of AiHSP70 gene.**
(DOCX)

Table S3 **Distribution of the AiHSP70 gene promoter haplotype in different populations.**
(DOCX)
