# Supplementary material for: The Polymorphism in the Promoter of HSP70 Gene Is Associated with Heat Tolerance of Two Congener Endemic Bay Scallops (Argopecten irradians irradians and A. i. concentricus)
Source: PLoS One. 2014 Jul 16;9(7):e102332. doi: 10.1371/journal.pone.0102332 (PMC4100766; doi:10.1371/journal.pone.0102332)
Supplement: Table S1 — The potential transcription start sites in the promoter of AiHSP70 gene. (DOCX) [file pone.0102332.s001.docx]

**Table S1.** The potential transcription start sites in the promoter of AiHSP70 gene.

| **Start** | **End** | **Promoter Sequence** | **Score** |
| --- | --- | --- | --- |
| -1116 | -1067 | taagtgcgcataaatactcgtcgcatagcgccgggaaccaagaacaaacc | 0.99 |
| -671 | -622 | aaatcgaccccattatataaccggttttgtttagttactcagtctttgcc | 0.91 |
| -424 | -375 | aagtatggtatataaatagagtgaacggaattccaagaataagtcacccc | 0.96 |
| -148 | -99 | ttttcatgacatatctaaaaggtgctagggaatggggtacactgtagatt | 0.92 |

Note: The gray shades indicate the potential transcription start site.
