# Supplementary material for: The Polymorphism in the Promoter of HSP70 Gene Is Associated with Heat Tolerance of Two Congener Endemic Bay Scallops (Argopecten irradians irradians and A. i. concentricus)
Source: PLoS One. 2014 Jul 16;9(7):e102332. doi: 10.1371/journal.pone.0102332 (PMC4100766; doi:10.1371/journal.pone.0102332)
Supplement: Table S2 — The D' value between the polymorphic loci in the promoter region of AiHSP70 gene. (DOCX) [file pone.0102332.s002.docx]

**Table S2.** The D' value between the polymorphic loci in the promoter region of AiHSP70 gene.

| loci | -1108 | -1107 | -999 | -967 | -894 | -480 | -408 | -204 | -83 | -28 |
| --- | --- | --- | --- | --- | --- | --- | --- | --- | --- | --- |
| -1248 | 0.619 | 0.619 | 0.599 | 0.641 | 0.700 | 0.779 | 0.632 | 0.199 | 0.606 | 0.380 |
| -1108 | - | 1.000 | 0.824 | 0.821 | 0.562 | 0.557 | 0.918 | 0.638 | 0.804 | 0.680 |
| -1107 | - | - | 0.824 | 0.821 | 0.562 | 0.557 | 0.918 | 0.638 | 0.804 | 0.680 |
| -999 | - | - | - | 0.666 | 0.340 | 0.592 | 0.518 | 0.621 | 0.734 | 0.338 |
| -967 | - | - | - | - | 0.470 | 0.523 | 0.629 | 0.880 | 0.948 | 0.391 |
| -894 | - |  | - | - | - | 0.422 | 0.783 | 0.067 | 0.396 | 0.514 |
| -480 | - | - | - | - | - | - | 0.318 | 0.277 | 0.500 | 0.136 |
| -408 | - | - | - | - | - | - | - | 0.400 | 0.778 | 1.000 |
| -204 | - | - | - | - | - | - | - | - | 0.776 | 0.991 |
| -83 | - | - | - | - | - | - | - | - | - | 0.766 |
