# Supplementary material for: The Polymorphism in the Promoter of HSP70 Gene Is Associated with Heat Tolerance of Two Congener Endemic Bay Scallops (Argopecten irradians irradians and A. i. concentricus)
Source: PLoS One. 2014 Jul 16;9(7):e102332. doi: 10.1371/journal.pone.0102332 (PMC4100766; doi:10.1371/journal.pone.0102332)
Supplement: Table S3 — Distribution of the AiHSP70 gene promoter haplotype in different populations. (DOCX) [file pone.0102332.s003.docx]

**Table S3.** The haplotype analysis of AiHSP70 polymorphic loci -1108,-1107,-83 and -28 in heat sensitive and heat resistant populations.

| **No.** | **Haplotype** | **Sen.**  **(freq)** | **Res.**  **(freq)** | **χ^2^ (*P*)** | | **OR [95% CI]** |
| --- | --- | --- | --- | --- | --- | --- |
| 1 | A T A A | 0.000 | 0.017 ↑ | | 0.53 (0.47) | 290.57 [11.794-7158.550] |
| 2 | A T T A | 0.016 | 0.05 ↑ | | 0.905 (0.34) | 3.217 [0.463-22.336] |
| 3 | A T T G * | 0.097 | 0.385 ↑ | | 14.09 (0.000176) | 5.845 [2.182-15.658] |
| 4 | C A T A | 0.032 | 0.111 ↑ | | 2.903 (0.09) | 3.754 [0.746-18.893] |
| 5 | C A A A | 0.564 | 0.419 ↓ | | 2.628 (0.105) | 0.556 [0.273-1.133] |
| 6 | C A T G * | 0.290 | 0.018 ↓ | | 17.59 (2.77e-005) | 0.045 [0.007-0.316] |

Note: OR, odds ratio; 95% CI, 95 percent confidence interval; *, *P* values < 0.05; ↑, increased; ↓, decreased.
